# Effects of phytosterol-rich foods on lipid profile and inflammatory markers in patients with hyperlipidemia: a systematic review and meta-analysis

**DOI:** 10.3389/fphar.2025.1619922

**Published:** 2025-07-02

**Authors:** Yihua Zhang, Qian Zhang, Xiumei Wang, Yatian Jia, Qingmei Niu, Shuo Ding, Wenjing Li

**Affiliations:** ^1^ School of Nursing, Shanxi University of Chinese Medicine, Yuci, China; ^2^ Department of Nursing, Shanxi Bethune Hospital, Shanxi Academy of Medical Sciences, Tongji Shanxi Hospital, Third Hospital of Shanxi Medical University, Taiyuan, China; ^3^ Department of Central Operating Room, Shanxi Bethune Hospital Shanxi Academy of Medical Sciences, Tongji Shanxi Hospital, Third Hospital of Shanxi Medical University, Taiyuan, China

**Keywords:** phytosterols, hyperlipidemia, blood lipids, inflammatory markers, systematic review

## Abstract

**Background:**

As naturally occurring compounds in plant-based foods, phytosterols have attracted attention for their lipid-modulating potential and proposed role as adjunctive therapies in managing hyperlipidemia. Nevertheless, conflicting evidence persists regarding their dual impact on dyslipidemia and subclinical inflammation.

**Objective:**

This systematic review aimed to assess the impact of phytosterol-rich foods on lipid metabolism and inflammatory responses in hyperlipidemic populations.

**Methods:**

A thorough literature search was performed across nine databases (including China National Knowledge Infrastructure Wanfang Data, VIP, SinoMed, PubMed, Cochrane Library, Embase, Scopus, Web of Science) from their inception up to 15 February 2025. Studies included were randomized controlled trials evaluating phytosterol interventions in adults with hyperlipidemia. The quality of the included studies was evaluated using the Cochrane Randomized Trial Risk Bias Tool, and Data analysis was performed using RevMan 5.4.

**Results:**

This study included 14 randomized controlled trials with a total of 1,088 participants. The pooled results demonstrated statistically significant reductions in total cholesterol (TC) levels (mean difference (MD) = −0.65, 95% CI −0.83 to −0.47, *P* < 0.00001) and low-density lipoprotein cholesterol (LDL-C) levels (MD = −0.52, 95% CI −0.66 to −0.38, *P* < 0.00001), along with a modest increase in high-density lipoprotein cholesterol (HDL-C) levels (MD = 0.08, 95% CI 0.05 to 0.10, *P* < 0.00001). No significant change was observed for C-reactive protein (CRP) levels (MD = −0.00, 95% CI −0.01 to 0.00, *P* = 0.32). Although a borderline significant reduction in triglycerides (TG) levels was noted (MD = −0.24, 95% CI −0.47 to −0.01, *P* = 0.04), this finding displayed considerable heterogeneity.

**Conclusion:**

Phytosterol intervention demonstrates significant efficacy in modulating atherogenic lipid profiles, such as TC and LDL-C, while also elevating HDL-C levels in individuals with hyperlipidemia. Yet, it fails to demonstrate anti-inflammatory activity as measured by CRP levels. The observed marginal TG-lowering effect should be interpreted with caution given substantial interstudy heterogeneity. Therefore, larger, metabolomics-inclusive studies are required for definitive conclusions and clinical guidance.

**Systematic Review Registration:**

https://www.crd.york.ac.uk/PROSPERO/#loginpage, identifier CRD420251002645.

## Introduction

Hyperlipidemia is a chronic metabolic disorder marked by lipid metabolism abnormalities. It is characterized by elevated levels of serum Total Cholesterol (TC), Triglycerides (TG), and Low-Density Lipoprotein cholesterol (LDL-C), while High-Density Lipoprotein cholesterol (HDL-C) levels are typically reduced (Yang et al., 2019). It is not only an important risk factor for atherosclerotic cardiovascular disease, but also contributes significantly to the global public health burden. According to the Centers for Disease Control and Prevention, approximately 53% of American adults have abnormal LDL-C levels, yet only 35% achieve recommended lipid targets. Moreover, approximately 31 million adults have TC levels exceeding 6.24 mmol/L, and those with uncontrolled hyperlipidemia face a 200% higher risk of cardiovascular events compared to the general population (Writing Group Members et al., 2016; Karr, 2017). In China, the prevalence of adult hyperlipidemia has surged to 35.6%, with younger populations increasingly affected (Wang et al., 2023). This has become an important area of focus for the prevention and control of chronic diseases.

Dyslipidemia is an independent risk factor for cardiovascular disease, posing a significant threat to human health if uncontrolled (Alloubani et al., 2021). In terms of disease classification, primary hyperlipidemia is mainly caused by inherited lipid metabolism defects, while secondary types are closely related to multi-system dysfunction caused by metabolic syndrome (such as obesity, diabetes, and hypertension) (Pan, 2023). While statins remain the cornerstone of treatment, approximately 50% of patients with familial hypercholesterolemia fail to reach target lipid levels even with high-dose therapy. Moreover, long-term use of these medications may cause serious adverse reactions, such as myalgia and rhabdomyolysis (Liu and Zhiping, 2023; Stroes et al., 2015). In addition, for patients with secondary hyperlipidemia complicated with multiple metabolic disorders, the clinical management needs to take into account the multi-target regulation of blood glucose and blood pressure, which further increases the complexity of treatment. Therefore, it is of great practical significance to explore safe and cost-effective auxiliary lipid-lowering strategies.

Phytosterols are natural triterpene compounds widely distributed in plant cell membranes, primarily existing in three chemical forms: free, esterified, and glycosidically-bound (Valitova et al., 2016). Its main sources are vegetable oils (such as canola oil, corn oil), nuts, seeds and legumes. Among them, β-sitosterol, campesterol, and stigmasterol collectively account for over 70% of the total phytosterols. Other common forms include spinach sterols, oat sterols, and sitostanol (Moreau et al., 2018). Recent studies have highlighted the diverse physiological functions of phytosterols, including lipid metabolism regulation, anti-inflammatory effects, and immune modulation (Nechchadi et al., 2024; Gagliardi et al., 2010). However, the clinical evidence regarding their lipid-lowering and anti-inflammatory effects remains inconsistent (Demonty et al., 2013; Garoufi et al., 2014; Rideout et al., 2015; Li and Xing, 2016; Ras et al., 2015; Sun et al., 2014; Dewi et al., 2024; Jie et al., 2022). Based on this, This systematic review and meta-analysis aims to clarify the impact of phytosterol supplementation on serum lipid profiles (TC, LDL-C, HDL-C, TG) and inflammatory markers (CRP) in hyperlipidemic populations, integrating evidence from randomized controlled trials (RCTs) to inform evidence-based dietary interventions.

## Materials and methods

This study was registered with PROSPERO (registration number: CRD420251002645) (https://www.crd.york.ac.uk/PROSPERO/#loginpage). During the preparation of this manuscript, it strictly abided by the guidelines outlined in the Primary Reporting Items for Systematic Reviews and Meta-Analyses (PRISMA) (Page et al., 2021).

### Inclusion criteria

Participants: Adults aged ≥18 years diagnosed with hyperlipidemia based on clinical criteria.

Interventions: Experimental groups received phytosterol-enriched food supplements (e.g., functional foods or nutritionally enhanced diets).

Control: The placebo group received inactive food matrices that were visually and indistinguishable in taste and appearance from the active interventions.

Outcome: The primary outcomes were lipid parameters, including Low-density lipoprotein cholesterol (LDL-C), Total cholesterol (TC), and the inflammatory marker C-reactive protein (CRP). The secondary outcomes included Triglycerides (TG) and High-density lipoprotein cholesterol (HDL-C). The Studies must provide data on at least one outcome parameter.

Study type: Randomized controlled trials (RCTs).

### Exclusion criteria

1. Studies on phytosterols combined with other drugs/nutrients intervention; 2. Studies that are replications of published studies; 3. Studies for which the full text or incomplete data were unavailable; 4. Reviews, conference abstracts, animal experimental studies, etc.; and 5. Studies on phytosterol pharmaceutical supplements.

### Search strategy

Two researchers independently searched nine databases (China National Knowledge Infrastructure (CNKI), Wanfang Data, VIP, SinoMed, PubMed, Cochrane Library, Embase, Scopus, Web of Science) from inception to 15 February 2025. A hybrid search strategy combining subject headings with free terms was employed. Search terms included: Phytosterols, phytosterol*, Plant sterol*, Phytostanol*, Sitosterol*, plant stanol*, sitostanol*, Campestanol*, Stigmasterol*, Stigmastanol*, brassicasterol*, Hypercholesterolemia, hyperlipoproteinemia, Hyperlipemia, dyslipidemias, randomized controlled trial, RCT, random, stud*. The language filter included both Chinese and English literature.

### Literature screening and data extraction

Two researchers independently conducted systematic search and literature management using EndNote 20, ensuring duplicate removal. Subsequently, the titles and abstracts were carefully reviewed to exclude irrelevant studies, followed by a thorough examination of the full texts to select relevant articles based on predefined inclusion and exclusion criteria. Data extraction was performed by two researchers independently, encompassing information such as the first author’s name, publication year, publication country, participants involved, sample size, intervention measures employed, intervention duration, outcome indicators assessed, among others.

### Literature quality assessment

The Cochrane Risk of Bias Tool was utilized to evaluate the methodological quality in seven domains: Randomization sequence generation; Allocation concealment; Blinding of participants and personnel; Blinding of outcome assessment; Incomplete outcome data; Selective reporting and Other bias. Studies were categorized into three quality levels (low, high, or unclear risk of bias) based on the risk-of-bias diagram. In case of discrepancies during the above process, a third researcher would act as an arbitrator to facilitate consensus-building.

### Evidence quality assessment

The certainty of the evidence was assessed using the Grading of Recommendations, Assessment, Development and Evaluations (GRADE) approach. The quality of evidence for Randomized Controlled Trials (RCTs) was initially rated as high according to the GRADE methodology. This rating may be downgraded to moderate, low, or very low if limitations are identified in any of the five domains: Risk of bias, Inconsistency, Indirectness, Imprecision, or Publication bias. Conversely, the evidence quality may be upgraded if large effect sizes or dose-response gradients are observed. In the event of disagreement during this assessment, a third researcher served as an arbitrator to reach a consensus.

### Data analysis methods

Meta-analysis was performed using Review Manager 5.4, adhering to the PRISMA guidelines for eligible studies. The results were presented as forest plots. Interstudy heterogeneity was assessed by the Cochrane heterogeneity test: a fixed-effect model was applied if *P* ≥ 0.1 and I^2^ ≤ 50%, otherwise a random-effects model was used for analysis. All outcome measures were standardized continuous variables, and effect sizes were reported as weighted mean differences (MD) with 95% confidence interval (CI). A *P*-value < 0.05 denoted statistical significance.

## Results

### Literature search results

**FIGURE 1:**
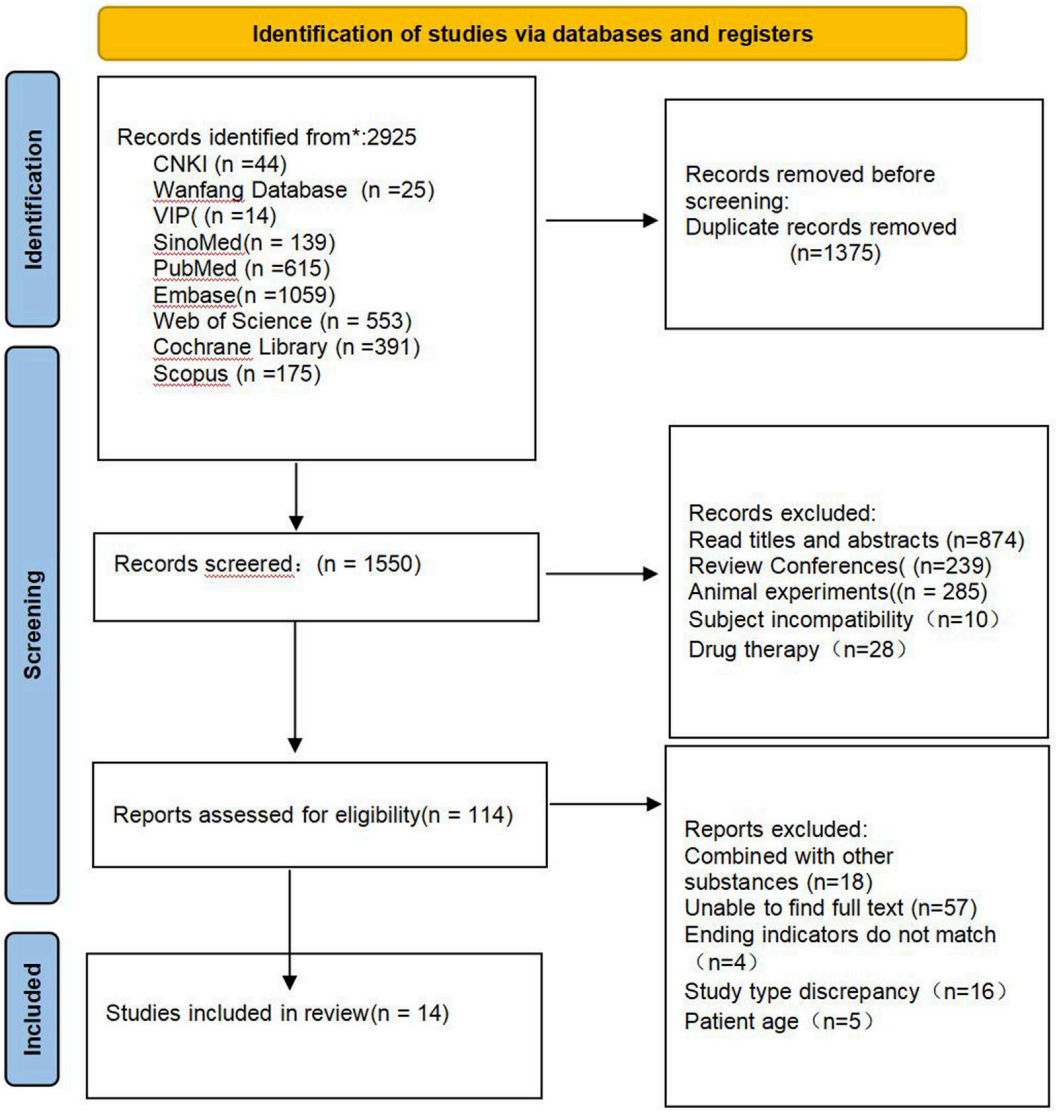
Literature screening process.

A total of 2,925 relevant literatures were obtained from the preliminary search database, and after excluding 1,375 duplicate literatures, 1,550 literatures remained. After preliminary screening by reading titles and abstracts, 1,436 articles that clearly did not meet the inclusion criteria were excluded, and 114 articles that might meet the inclusion criteria were obtained. After further reading the full text, 100 ineligible literatures were excluded, and 14 literatures were finally included (Dewi et al., 2024; Wang et al., 2015; Orem et al., 2017; Eady et al., 2011; Lestiani et al., 2018; Athyros et al., 2011; Vásquez-Trespalacios and Romero-Palacio, 2014; Hallikainen et al., 2013; Oliveira et al., 2020; Theuwissen et al., 2009; Kriengsinyos et al., 2015; Buyuktuncer et al., 2013; Cicero et al., 2023; Dong et al., 2016). The literature screening process and results are shown in Figure 1.

### Basic characteristics of the included studies

This study incorporated 14 randomized controlled trials involving 1,088 hyperlipidemic patients. All studies described the baseline characteristics of the two groups, and these groups were comparable. Furthermore, all studies reported the outcome measures. Detailed baseline information for the included studies is presented in Table 1.

**TABLE 1:** Basic characteristics of the included literature.

First author, publication year	Country	Health condition	Sample size (T/C)	Sex (male/female) (T/C)	Intervention (daily dose)	Control intervention	Duration	Outcomes	Outcomes detail
Dewi et al. (2024)	Indonesia	Hyperlipidemia	100 (50/50)	T:14/36 C:10/40	Plant sterol-enriched palm oil (2 g/day)	Placebo palm oil	8 weeks	①:a,b,c,d ②:e	TC, LDL-C ↓; TC/HDL-C and LDL-C/HDL-C ratios improved (trends). No change in CRP.
Cicero et al. (2023)	Italy	Hypercholesterolemia	99 (49/50)	T:15/34 C:15/35	Plant sterol-enriched RTD supplement (2.5 g/day)	Placebo drink	3 weeks	①:a,b,c,d	TC, LDL-C ↓
Lestiani et al. (2018)	Indonesia	Hypercholesterolemia	88 (43/45)	T:16/27 C:22/23	Plant sterol-enriched milkshake (2 g/day)	Plain milkshake	4 weeks	①:a,b,c,d	TC, LDL-C ↓
Orem et al. (2017)	Turkey	hypercholesterolemia	66 (32/34)	T:24/8 C:23/11	Plant sterol-enriched black tea (2 g/day)	Placebo tea	4 weeks	①:a,b,c,d ②:e	TC, LDL-C ↓. No changes in HDL-C, TG, or inflammatory markers
Dong et al. (2016)	China	Hyperlipidemia	137 (69/68)	Unreported	Plant sterol-enriched soy milk (2 g/day)	Plain soy milk	6 months	①:a,b,c,d	TC, LDL-C, non-HDL-C ↓
Kriengsinyos et al. (2015)	Thailand	hypercholesterolemia	119 (59/60)	T:15/44 C:14/46	Plant sterol-enriched biscuits (2 g/day)	Placebo biscuits	4 weeks	①:a,b,c,d	TC, LDL-C ↓; LDL/HDL ratio improved
Hallikainen et al. (2013)	Finland	Hypercholesterolemia	56 (27/29)	T:4/23 C:7/22	Plant sterol-enriched soy drink (2.7 g/day)	Placebo soy drink	4 weeks	①:a,b,c,d ②:e	TC, LDL-C ↓. No changes in HDL-C or TG.
Buyuktuncer et al. (2013)	Turkey	hypercholesterolemia	51 (23/28)	T:12/11 C:14/14	Plant sterol-enriched yogurt (1.9 g/day)	Placebo yogurt	4 weeks	①:a,b,c,d	TC, LDL-C ↓; ox-LDL ↓
Eady et al. (2011)	New Zealand	hypercholesterolemia	80 (40/40)	T:10/30 C:17/23	Plant sterol-enriched spread (2 g/day)	Placebo spread	4-week	①:a,b,c,d	TC, LDL-C↓, HDL-C↑
Athyros et al. (2011)	Greece	Hypercholesterolemia	100 (50/50)	T:24/26 C:24/26	Plant sterol ester-enriched spread (2 g/day)	Placebo spread	4 months	①:a,b,c,d ②:e	TC, LDL-C ↓; hsCRP ↓
Oliveira et al. (2020)	Brazil	Hypercholesterolemia	38 (38/38)	7M/31F	Plant sterol-enriched soy milk (1.6 g/day)	Plain soy milk	4 weeks	①:a,b,c,d ②:e	TC, LDL-C ↓. No changes in HDL-C or CRP. Baseline high-LDL subgroup showed TG ↓ trend
Vásquez-Trespalacios and Romero-Palacio (2014)	Colombia	Hypercholesterolemia	40 (40/40)	10M/30F	Plant sterol-enriched yogurt drink (4 g/day)	Placebo yogurt drink	4 weeks	①:a,b,c,d	TC, LDL-C ↓. No significant changes in HDL-C or TG.
Theuwissen et al. (2009)	Netherlands	Hyperlipidemia	14 (14/14)	8M/6F	Plant sterol-enriched margarine (2.5 g/day)	Placebo margarine	3 weeks	①:a,b,c,d	TC, LDL-C, TG ↓
Wang et al. (2015)	China	Hyperlipidemia	100 (50/50)	T: 28/22 C: 24/26	Plant sterol-enriched milk (2.125 g/day)	Placebo milk	45 days	①: b, c, d	TC, TG ↓.HDL-C↑

①Blood lipid: a. LDL-C, low-density lipoprotein cholesterol; b. TC, total cholesterol; c. HDL-C, high-density lipoprotein cholesterol; d. TG, triglyceride; ②inflammatory indicators: e. CRP, C-reactive protein.

### Methodological quality of the included studies

**FIGURE 2:**
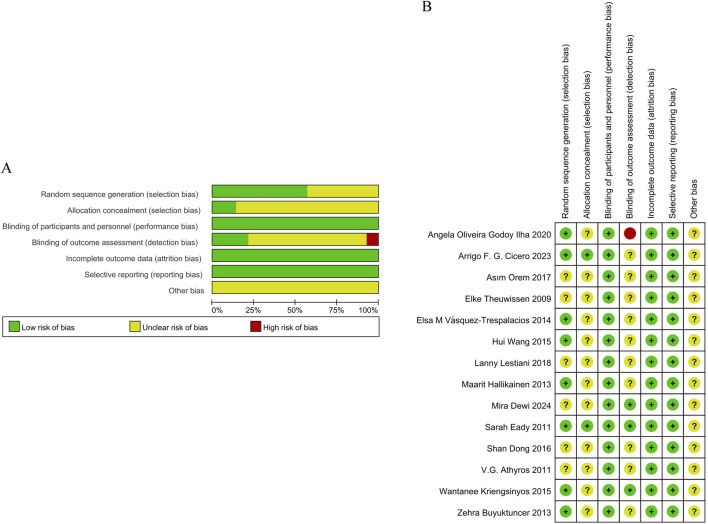
**(A)** Risk of bias graph and **(B)** Risk of bias summary.

The methodological quality of the 14 included studies was systematically assessed using the Cochrane Risk of Bias tool, revealing heterogeneous risk profiles across evaluation domains. Eight studies (Wang et al., 2015; Eady et al., 2011; Vásquez-Trespalacios and Romero-Palacio, 2014; Hallikainen et al., 2013; Oliveira et al., 2020; Kriengsinyos et al., 2015; Buyuktuncer et al., 2013; Cicero et al., 2023) demonstrated low risk through explicit random number table or stratified randomization methods, while six studies lacked sufficient detail on randomization processes and were classified as unclear risk. Allocation concealment was adequately described in two studies (Eady et al., 2011; Cicero et al., 2023), earning them low risk ratings, but unclear in the rest. Regarding blinding, all studies reported participant blinding with low risk; three studies (Dewi et al., 2024; Eady et al., 2011; Kriengsinyos et al., 2015) further blinded outcome assessors, reinforcing their low risk classification. Conversely, 10 studies omitted details regarding blinding, resulting in unclear risk, and one study (Oliveira et al., 2020) failed to blind statisticians, resulting in a high-risk designation. Data integrity was generally robust, as all studies documented dropout rates and reasons, yielding low risk for bias. Reporting bias remained unclear due to insufficient evidence of selective outcome reporting, and no studies described quality control protocols. These findings are comprehensively presented in Figures 2A,B.

### Quality of evidence

According to the GRADE assessment, the certainty of evidence was rated as low for TG, LDL-C, HDL-C, and TC outcomes. For CRP, the evidence was deemed very low certainty. These downgrades primarily stem from serious limitations in risk of bias (due to inadequate randomization, allocation concealment, and blinding) and imprecision (attributable to small sample sizes and wide confidence intervals). The evaluation details are in Table 2.

**TABLE 2:** Quality assessment.

Quality assessment							Effect	Quality	Importance
No of studies	Design	Risk of bias	Inconsistency	Indirectness	Imprecision	Other considerations	Rate (95% CI)		
TC (Better indicated by lower values)									
14	Randomised trials	Serious1	No serious inconsistency	No serious indirectness	Serious2	None	MD 0.65 lower (0.83–0.47 lower)	⊕⊕ΟΟ Low	Critical
TG (Better indicated by lower values)									
14	Randomised trials	Serious1	No serious inconsistency	No serious indirectness	Serious2	None	MD 0.24 lower (0.47–0.01 lower)	⊕⊕ΟΟ Low	Critical
LDL-C (Better indicated by lower values)									
13	Randomised trials	Serious1	No serious inconsistency	No serious indirectness	Serious2	None	MD 0.52 lower (0.66–0.38 lower)	⊕⊕ΟΟ Low	Critical
HDL-C (Better indicated by lower values)									
14	Randomised trials	Serious1	No serious inconsistency	No serious indirectness	Serious2	None	MD 0.08 higher (0.05–0.1 higher)	⊕⊕ΟΟ Low	Critical
CRP (Better indicated by lower values)									
5	Randomised trials	Serious1	No serious inconsistency	No serious indirectness	Very serious3	None	MD 0.01 lower (0.02 lower to 0.01 higher)	⊕ΟΟΟ Very low	Critical

Serious1: The included studies were assessed as having a high risk of bias due to deficiencies in randomization, allocation concealment, and blinding.

Serious2: The included studies were limited by small sample sizes.

Serious3: The included studies were limited by small sample sizes, resulting in wide confidence intervals that indicate imprecision of effect estimates.

### Effects of phytosterol-rich foods on patients with hyperlipidemia

#### Effects of phytosterol-rich foods on TC in patients with hyperlipidemia

**FIGURE 3:**
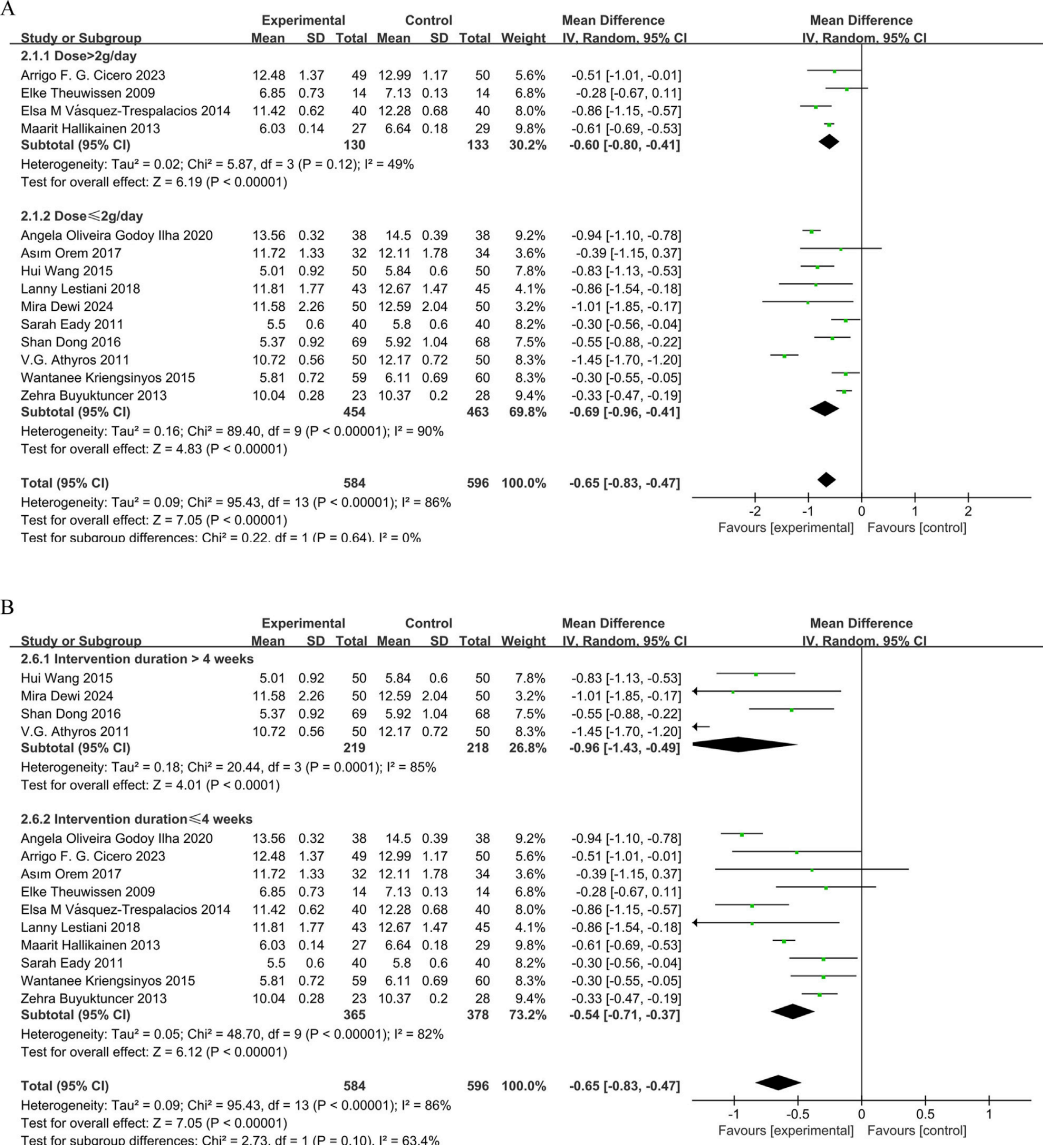
**(A)** Meta-analysis results of TC change in included trials, stratified by intervention dose and **(B)** Meta-analysis results of TC change in included trials, stratified by intervention duration.

14 studies (Dewi et al., 2024; Wang et al., 2015; Orem et al., 2017; Eady et al., 2011; Lestiani et al., 2018; Athyros et al., 2011; Vásquez-Trespalacios and Romero-Palacio, 2014; Hallikainen et al., 2013; Oliveira et al., 2020; Theuwissen et al., 2009; Kriengsinyos et al., 2015; Buyuktuncer et al., 2013; Cicero et al., 2023; Dong et al., 2016) demonstrated that phytosterol interventions significantly reduced TC levels (MD = −0.65, 95% CI −0.83 to −0.47, *P* < 0.00001). However, substantial heterogeneity (*P* < 0.00001, I^2^ = 86%) was observed. Subgroup analysis indicated that intervention duration exhibited a significant dose-independent effect on TC reduction (P < 0.05). But no meaningful interaction between phytosterol dosage and TC outcomes. The forest plots showed that although there was significant heterogeneity, the overall effect size remained stable. See Figures 3A,B.

#### Effects of phytosterol-rich foods on LDL-C in patients with hyperlipidemia

**FIGURE 4:**
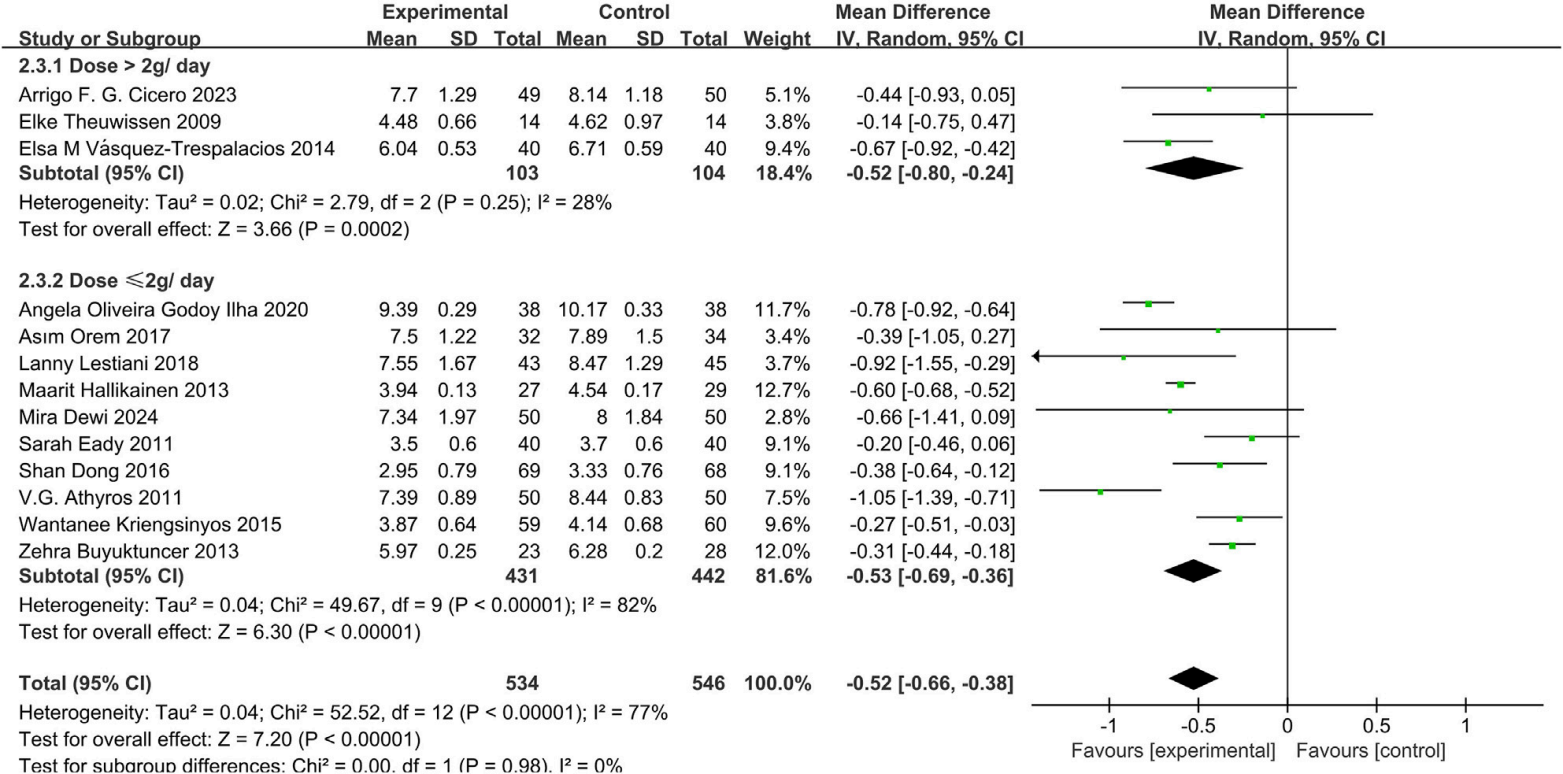
Meta-analysis results of LDL-C change in included trials.

13 studies (Dewi et al., 2024; Orem et al., 2017; Eady et al., 2011; Lestiani et al., 2018; Athyros et al., 2011; Vásquez-Trespalacios and Romero-Palacio, 2014; Hallikainen et al., 2013; Oliveira et al., 2020; Theuwissen et al., 2009; Kriengsinyos et al., 2015; Buyuktuncer et al., 2013; Cicero et al., 2023; Dong et al., 2016) demonstrated that phytosterols could significantly reduce LDL-C levels (MD = −0.52, 95% CI −0.66 to −0.38, *P* < 0.00001), with high heterogeneity (*P* < 0.00001, I^2^ = 77%). Subgroup analysis showed no significant dose interaction and indicated stable overall effect sizes despite high heterogeneity. See Figure 4.

#### Effects of phytosterol-rich foods on HDL-C in patients with hyperlipidemia

**FIGURE 5:**
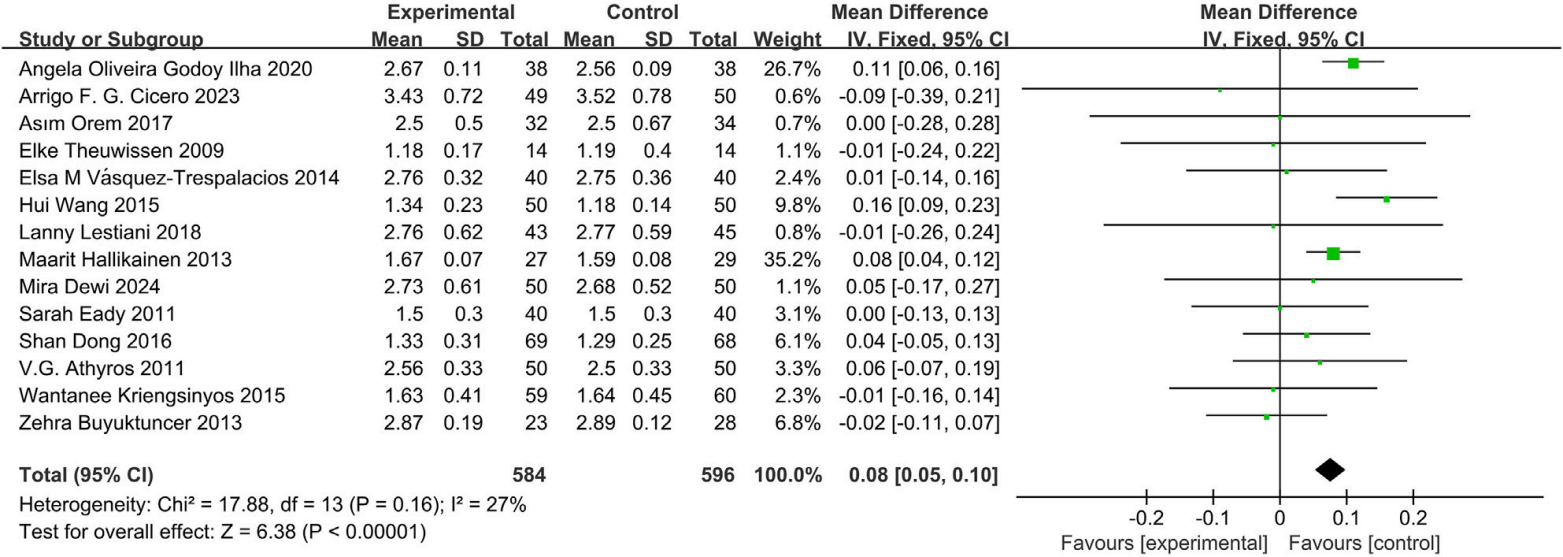
Meta-analysis results of HDL-C change in included trials.

14 studies (Dewi et al., 2024; Wang et al., 2015; Orem et al., 2017; Eady et al., 2011; Lestiani et al., 2018; Athyros et al., 2011; Vásquez-Trespalacios and Romero-Palacio, 2014; Hallikainen et al., 2013; Oliveira et al., 2020; Theuwissen et al., 2009; Kriengsinyos et al., 2015; Buyuktuncer et al., 2013; Cicero et al., 2023; Dong et al., 2016) demonstrated that phytosterols could significantly reduce HDL-C levels, with a statistically significant difference (MD = 0.08, 95% CI 0.05 to 0.10, *P* < 0.00001). See Figure 5.

#### Effect of phytosterol-rich foods on TG in patients with hyperlipidemia

**FIGURE 6:**
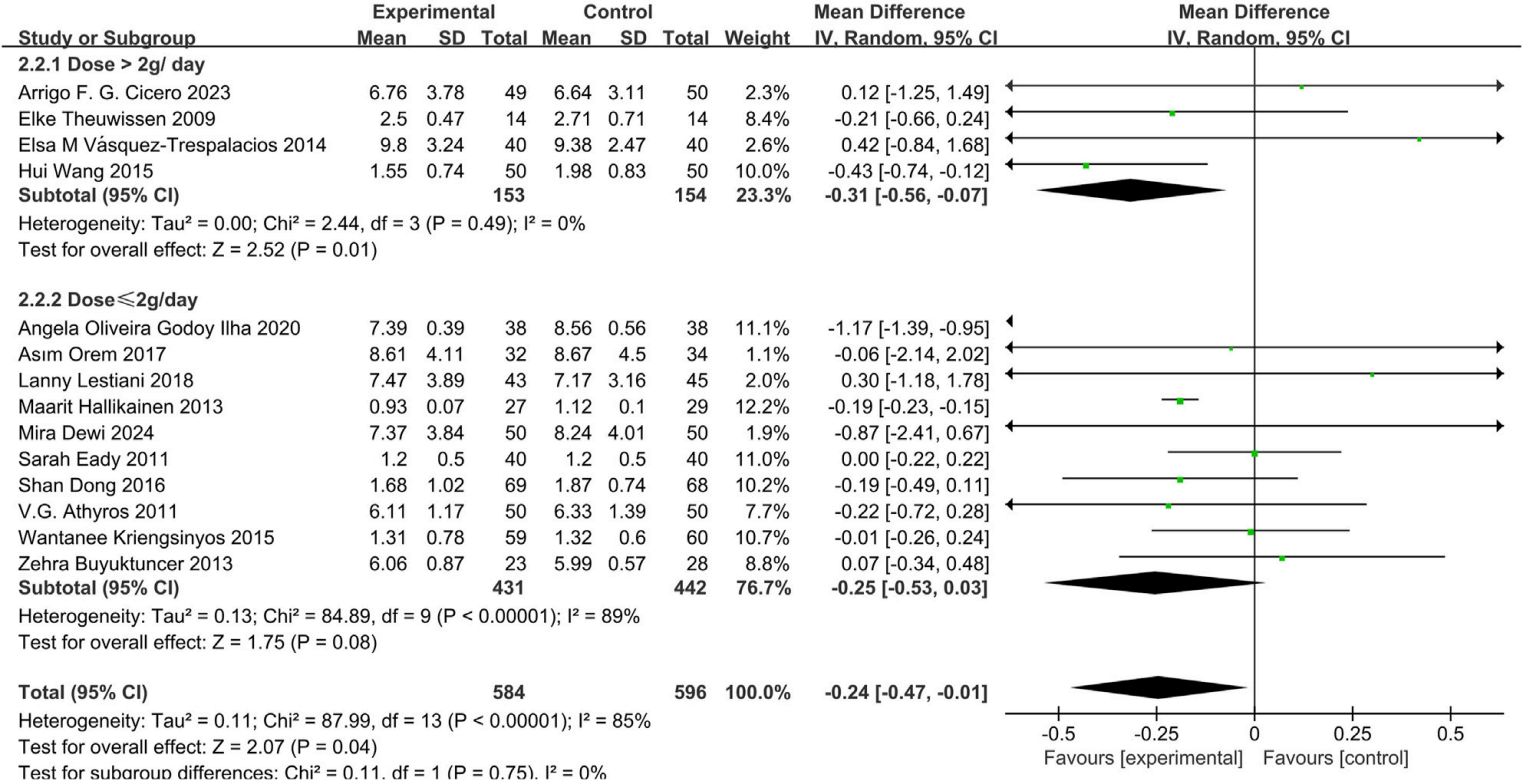
Meta-analysis results of TG change in included trials.

14 studies (Dewi et al., 2024; Orem et al., 2017; Eady et al., 2011; Lestiani et al., 2018; Athyros et al., 2011; Vásquez-Trespalacios and Romero-Palacio, 2014; Hallikainen et al., 2013; Oliveira et al., 2020; Theuwissen et al., 2009; Kriengsinyos et al., 2015; Buyuktuncer et al., 2013; Cicero et al., 2023; Dong et al., 2016) demonstrated that phytosterols reduced TG levels (MD = −0.24, 95% CI −0.47 to −0.01, *P* = 0.04). Heterogeneity analysis showed significant inter-study heterogeneity (*P* = 0.04, I^2^ = 85%). Subgroup analysis revealed a significant decrease in TG levels in the high-dose group (>2 g/day) (MD = −0.31, 95% CI −0.56 to −0.07, *P* = 0.01), but not in the low-dose group (≤2 g/day) (MD = −0.25, 95% CI −0.53 to 0.03, *P* = 0.08). The overall effect size was statistically significant. See Figure 6.

#### Effect of phytosterol-rich foods on CRP in patients with hyperlipidemia

**FIGURE 7:**
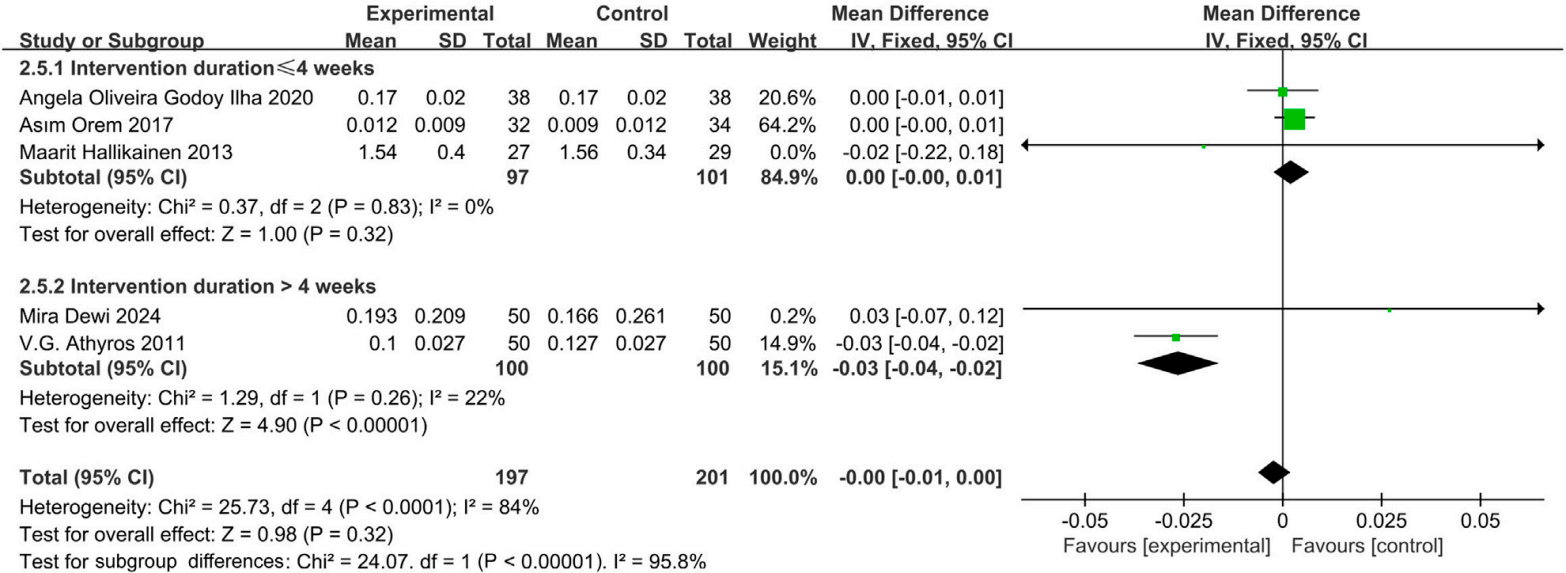
Meta-analysis results of CRP change in included trials.

Five studies (Orem et al., 2017; Athyros et al., 2011; Hallikainen et al., 2013; Oliveira et al., 2020; Theuwissen et al., 2009) indicated that phytosterols had no significant effect on CRP levels (MD = −0.00, 95% CI −0.01 to 0.00, *P* = 0.32). Heterogeneity analysis showed significant inter-study heterogeneity (*P* = 0.06, I^2^ = 75%). Subgroup analysis showed a significant reduction in CRP levels when intervention duration >4 weeks (MD = −0.03, 95% CI −0.04 to −0.02, *P* < 0.00001), with no significant change in shorter interventions (MD = 0.00, 95% CI −0.00 to 0.01, *P* = 0.32). The forest plots suggested that although there was heterogeneity, the overall effect size was not significant. See Figure 7.

### Adverse reactions

Only one of the included studies (Lestiani et al., 2018) on smoothie drinks reported phytosterol-related gastrointestinal adverse effects. There were no differences between the study groups in the symptoms of mild and transient changes in stool characteristics, upper abdominal discomfort, abdominal distension, increased flatulence and dyspepsia.

### Sensitivity analysis and publication bias

The pooled effect sizes for TC, LDL-C, HDL-C, and CRP remained statistically significant (*P* < 0.05) and unchanged after excluding any single study. The confidence intervals consistently stayed within the clinical significance threshold, indicating robustness of the results. For TG, removing the study (Oliveira et al., 2020) substantially reduced heterogeneity (I^2^ from 85% to 0%) and yielded a more precise estimate (MD = −0.18, 95% CI −0.22 to −0.14, *P* < 0.00001). The results remained stable. This adjustment was attributed to the extreme effect size in the excluded study, which may reflect methodological differences or data distribution variations compared to other trials.

**FIGURE 8:**
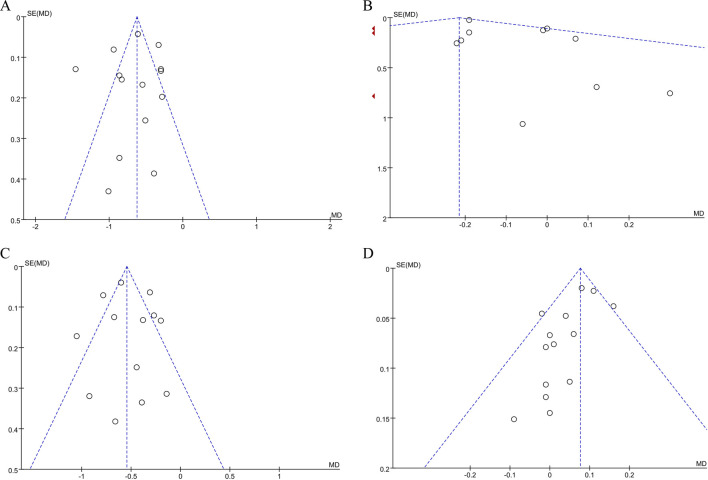
**(A)** Publication bias assessment: funnel plot for TC. **(B)** Publication bias assessment: funnel plot for TG. **(C)** Publication bias assessment: funnel plot for LDL-C. **(D)** Publication bias assessment: funnel plot for HDL-C.

Funnel plot analysis was performed on TC, TG, LDL-C and HDL-C of ≥10 included studies: The funnel plot of TC and LDL-C was symmetrical, and the study points were distributed symmetrically on both sides of the axis, with a small publication bias. However, the funnel plots of TG and HDL-C showed funnel asymmetry, suggesting potential publication bias and heterogeneity. See Figure 8A–B.

## Discussion

A total of 14 randomized controlled trials involving 1,088 patients were included in this study to evaluate the effects of phytosterols on blood lipids and inflammatory markers in patients with hyperlipidemia.

### Effects of phytosterol-rich foods on blood lipids and inflammatory markers in patients with hyperlipidemia

This meta-analysis found phytosterol consumption significantly reduced TC and LDL-C while increasing HDL-C. Effects on TG and CRP, however, were inconsistent across studies (Amir Shaghaghi et al., 2013; Jie and Kang, 2017; Gao et al., 2023; Jm et al., 2009). While two studies reported no significant impact on TG and HDL-C (Jie and Kang, 2017; Gao et al., 2023), this study confirmed significant TG reduction and HDL-C elevation in hyperlipidemic patients.

A dose-dependent TG reduction was observed. Subgroup analysis indicated that high-dose phytosterol intake (>2 g/day) significantly lowered TG levels (MD = −0.31, 95% CI −0.56 to −0.07, *P* = 0.01), particularly in individuals with baseline TG > 150 mg/dL (Orem et al., 2017; Theuwissen et al., 2009). In contrast, the low-dose group (≤2 g/day) and overall combined analysis showed non-significant trends (MD = −0.25, 95% CI −0.53 to 0.03, P = 0.08). A dose-effect correlation was confirmed with phytosterol-fortified milk (Wang et al., 2015). However, the Egger test, conducted using Stata17 software to explore publication bias, suggested potential publication bias (*P* = 0.0154), and the effect size remained stable (Hedges’s g = −0.387, 95% CI −0.794 to 0.02) after trim-and-fill analysis, possibly due to small sample sizes and methodological heterogeneity. Thus, current evidence is insufficient to conclusively support the TG-lowering effect of phytosterols, and further large-scale studies are needed.

Regarding HDL-C, studies (Sun et al., 2014; Wang et al., 2015) reported significant increases, whereas another study (Ras et al., 2015) observed no significant changes. A meta-analysis (Jie and Kang, 2017) suggested that phytosterols had no effect on HDL-C in either healthy individuals or hyperlipidemic populations. Indicating that population-specific metabolic characteristics may influence the observed outcomes. The Egger test did not detect significant publication bias (*P* = 0.6054).

Several studies (Gagliardi et al., 2010; Athyros et al., 2011; Devaraj et al., 2006) have shown that phytosterols significantly reduce CRP levels, suggesting potential anti-inflammatory mechanisms via antioxidant pathways. However, current study found no significant overall effect of phytosterols on inflammation (MD = −0.00, 95% CI −0.01 to 0.00, *P* = 0.32). Subgroup analysis revealed that intervention durations >4 weeks were associated with significantly lower CRP levels (MD = −0.03, 95% CI −0.04 to −0.02, *P* < 0.00001), indicating duration-dependent modulation of inflammation. Further research on mechanisms and clinical relevance is needed.

Phytosterols feature a sterane ring system with a C-24 methyl or ethyl group, a C-3 hydroxyl group, and one to two double bonds in ring B (Khallouki et al., 2024). Resembling cholesterol structurally, they reduce cholesterol via a multi-tiered “gut-liver-fat” regulatory axis. Their effectiveness hinges on the C-24 substituent and ring saturation. Hydrophobic C-24 groups boost efficacy, like in 4-desmethyl sterols (sitosterol, stigmasterol) which activate liver X receptors (LXRα) and upregulate ABCG5/G8 (He et al., 2018). Sitostanol, owing to its saturated structure, exhibits minimal absorption and persists within intestinal micelles and emulsions, thereby continuously disrupting cholesterol solubilization to exert hypocholesterolemic effects (Ikeda and Sugano, 1983).

In the intestine, phytosterols branched hydroxyl groups enhance lipid solubility and displace dietary cholesterol from bile acid micelles, reducing cholesterol absorption by 30%–50% (Nechchadi et al., 2024; Dumolt and Rideout, 2017; Xue et al., 2019). Key mechanisms involve the Niemann-Pick C1-Like1 (NPC1L1) protein: phytosterols enter intestinal cells via NPC1L1 but inhibit its cholesterol uptake function. They also suppress the acyl-CoA: cholesterol acyltransferase (ACAT) enzyme, reducing chylomicron formation needed for cholesterol transport into the bloodstream (Paalvast et al., 2017; Liang et al., 2011; Alphonse and Jones, 2016). Furthermore, ABCG5/G8 transporters pump absorbed phytosterols back into the intestine, limiting their circulation and further reducing cholesterol absorption (Ghosh et al., 2021).

Within the liver, phytosterols trigger LXRα, a transcription factor controlling cholesterol levels. Activated LXRα increases the expression of transporters like ABCA1 and ABCG5/G8, promoting cholesterol excretion into bile It also stimulates bile acid synthesis via cytochrome P450 7A1 (CYP7A1), creating a feedback loop that lessens cholesterol reabsorption (He et al., 2013; Pannu et al., 2013). Furthermore, phytosterols interfere with the activation of SREBP2, a protein crucial for making cholesterol. This reduces the activity of HMG-CoA reductase, a key enzyme in cholesterol biosynthesis (Alphonse and Jones, 2016; Batta et al., 2006).

Regarding blood fats, phytosterols lower liver TG production by blocking fat-making enzymes and help break down TG by boosting an enzyme called lipoprotein lipase (Nechchadi et al., 2024). Phytosterols also reduce cholesterol absorption and synthesis, thereby prompting an upsurge in endogenous cholesterol production and augmenting the hepatic uptake of plasma LDL-C. enhancing its clearance and lowering plasma concentration (Poli et al., 2021).

Recent studies Indicated that non-nutrient bioactive compounds, like polyphenols and phytosterols, found in plant foods, have therapeutic potential for chronic diseases. These compounds possess antioxidant, anti-inflammatory, and other health-promoting properties, acting through mechanisms distinct from conventional nutrients. (Zhu et al., 2023). Researchers have analyzed common healthy dietary patterns such as the Mediterranean and Japanese diets, highlighting the significance of non-nutrients in disease prevention and health promotion. Based on this analysis, “theoretical model of family nurse diet therapy” has been proposed, highlighting the potential benefits of non-nutrients within dietary interventions (Han et al., 2023). This theory states that non-nutrients aid in the treatment of chronic diseases through their anti-inflammatory, antioxidant, and metabolic regulatory properties.

Study (Jia et al., 2024a) confirmed that polyphenol-rich non-nutrient foods can improve metabolic abnormalities in patients with hyperlipidemia. Furthermore, a systematic review (Jia et al., 2024b) on patients with coronary heart disease demonstrated that polyphenol-rich seed foods can significantly reduce blood lipid and inflammation levels in patients with coronary heart disease. It is worth noting that as an important non-nutrient, phytosterols exemplify this theory by integrating cholesterol-lowering, anti-inflammatory, and antioxidant effects. While humans lack endogenous synthesis pathways for these sterols, dietary supplementation confers measurable health benefits (Shi et al., 2023).

Current guidelines recommend ≥2 g/day of phytosterols to achieve LDL-C reductions (Yuen et al., 2019), with some trials suggesting enhanced efficacy at doses >2.5–3 g/day (Fontané et al., 2023). However, the efficacy of phytosterols is influenced by several factors, including the food matrix, dosage, and intervention duration (Abumweis et al., 2008). For instance, oil-based carriers such as palm oil and butter may enhance the bioavailability of phytosterols due to their solubility advantages, while water-soluble substrates like soy milk and yogurt show relatively limited effects (Dewi et al., 2024). Despite these differences, one study has reported that the lipid-lowering efficacy of phytosterols is independent of the food substrate (American Heart Association Nutrition Committee et al., 2006). Long-term intake of phytosterols has not been associated with serious adverse reactions, but the bioavailability differences among various substrates should be considered. Existing evidence indicates that phytosterols offer potential health benefits in dyslipidemia populations. However, the clinical translation of their anti-inflammatory and lipid-lowering mechanisms requires further verification through large-scale, high-quality studies.

### Practical inspirations

Food is increasingly recognized as a frontline therapy for chronic disease management, offering advantages in safety, accessibility, and sustainability over pharmaceuticals (Moreno-Fernández et al., 2018). With the in-depth research on nutrition and chronic diseases, “Functional food” has attracted much attention as a new strategy for disease prevention and treatment, which has health-promoting and potential therapeutic use for chronic diseases (Malaguti et al., 2014). As natural, safe, and cost-effective dietary components, phytosterols exemplify this approach. Evidence indicates that dietary adjustments increasing plant sterol intake can effectively reduce chronic disease risk and improve health (Nattagh-Eshtivani et al., 2022).

In clinical practice, plant sterol intake strategies should be individualized to patients’ lipid profiles and dietary preferences. For patients with elevated baseline lipids, fortified foods (e.g., margarine, cereals) offer standardized dosing and effortless dietary integration. Conversely, those with lower lipid levels or seeking holistic nutrition benefit more from natural sources like nuts and vegetable oils, which simultaneously provide essential fatty acids and fiber while increasing sterol intake. Healthcare professionals must therefore tailor sterol source and dosage to each patient’s metabolic status and health objectives to optimize hyperlipidemia prevention and management.

### Strengths and limitations of the study

The studies included in this study were all randomized controlled trials that had been assessed by Cochrane risk of bias, covering 11 countries, and the evidence level was high. As natural dietary components, plant sterols have a significantly lower incidence of adverse events than chemical drugs, which is in line with the concept of “homologous medicine and food” and has prominent clinical safety advantages. This study provides robust evidence that phytosterols not only lower lipid levels but also modulate TG levels in a dose-dependent manner in hyperlipidemic patients, with a clinically significant reduction in TG requiring an intake >2 g/day. Secondly, it reveals that the duration of intervention >4 weeks is crucial for its anti-inflammatory effects, significantly reducing CRP levels. Furthermore, within the framework of “Functional food” and non-nutrient therapy, phytosterols have been shown to be a safe, cost-effective, and health-promoting intervention, offering a new approach to the nutritional management of chronic diseases.

This study also has some limitations. The scope of study search is limited to Chinese and English studies, and high-quality studies in non-English/non-Chinese regions may be missed. Most of the included studies were short - and medium-term trials, and lack of long-term efficacy and safety tracking of phytosterols may affect the results of phytosterols on blood lipids and inflammatory indicators. Subgroup analysis of certain outcome indicators, such as TG is limited by small sample size, and the results may be biased. In the future, multi-language, multi-center, long-term randomized controlled trials should be carried out, and personalized dosing strategies should be explored.

## Conclusion

In conclusion, phytosterols supplementation can improve the levels of LDL-C, TC, and HDL-C in patients with hyperlipidemia, but has no significant effect on CRP level. The underlying mechanisms may involve dual regulation of cholesterol absorption and anti-inflammatory pathways. In practice, researchers can design phytosterol-rich dietary plans tailored to patients’ energy needs and individual factors. Patients can choose food flexibly according to their own economic situation and dietary preferences. Future research should focus on large-scale, long-term studies to further elucidate the clinical significance and anti-inflammatory mechanisms of phytosterols. Thereby providing a robust scientific basis for clinical decision-making.

## Data Availability

The original contributions presented in the study are included in the article/Supplementary Material, further inquiries can be directed to the corresponding author.

## Abbreviations

HDL-C, high-density lipoprotein cholesterol; LDL-C, low-density lipoprotein cholesterol; TC, total cholesterol; TG, triglyceride; MD, Mean Difference; 95% CI, 95% Confidence Interval; CRP, C-reactive protein.
