# Supplementary material for: Effects of phytosterol-rich foods on lipid profile and inflammatory markers in patients with hyperlipidemia: a systematic review and meta-analysis
Source: Front Pharmacol. 2025 Jul 2;16:1619922. doi: 10.3389/fphar.2025.1619922 (PMC12263549; doi:10.3389/fphar.2025.1619922)
Supplement: Supplementary file 1 [file DataSheet1.docx]

The search terms include: Phytosterols, phytosterol*, Plant sterol*, Phytostanol*, Sitosterol*, plant stanol*, sitostanol*, Campestanol*, Stigmasterol*, Stigmastanol*, brassicasterol*, Hypercholesterolemia, hyperlipoproteinemia, Hyperlipemia, dyslipidemias, randomized controlled trial, RCT, random, stud*.

**Specific search strategy：**

Nine databases, including Chinese National Knowledge Infrastructure(CNKI), VIP Database, China Biomedical Literature Database(Sinomed), Wanfang Database, Pubmed, Cochrane Library, Embase, Scopus and Web of Science, were searched using the combination of MeSH terms and free words. The retrieval period was from the inception of the database to February 15, 2025.

1. **In Chinese databases, the search was conducted considering the CNKI database as an example of a retrieval strategy:**

**(主题:植物甾醇) OR (主题:植物甾烷醇 + 植物甾烷素 + 谷甾醇 + 谷甾烷醇 + 菜油甾醇 + 豆甾醇 + 豆甾烷醇 + 油菜甾醇) AND (主题:高脂血症) OR (主题:高胆固醇血症 + 高甘油三酯血症 + 高胆甾醇血症 + 血脂异常) AND (主题:随机对照试验) OR (主题:随机 + 研究)**

1. **In Chinese databases, the search was conducted considering the Wanfang database as an example of a retrieval strategy:**

**题名或关键词:(植物甾醇 or 植物甾烷醇 or 植物甾烷素 or 谷甾醇 or 谷甾烷醇 or 菜油甾醇 or 豆甾醇 or 豆甾烷醇 or 油菜甾醇) and 题名或关键词:(高脂血症 or 高胆固醇血症 or 高甘油三酯血症 or 高胆甾醇血症 or 血脂异常) and 题名或关键词:(随机对照试验 or 随机 or 研究)**

1. **In Chinese databases, the search was conducted considering the VIP database as an example of a retrieval strategy:**#1 AND #2 AND #3

#1**题名或关键词：植物甾醇 or 植物甾烷醇 or 植物甾烷素 or 谷甾醇 or 谷甾烷醇 or 菜油甾醇 or 豆甾醇 or 豆甾烷醇 or 油菜甾醇**

#2**题名或关键词：高脂血症 or 高胆固醇血症 or 高甘油三酯血症 or 高胆甾醇血症 or 血脂异常**

#3**题名或关键词：随机对照试验 or 随机 or 研究**

1. **In Chinese databases, the search was conducted considering the Sinomed database as an example of a retrieval strategy:**

**(("植物甾醇"[常用字段:智能] OR "植物甾烷醇"[常用字段:智能] OR "菜油甾醇"[常用字段:智能] OR "植物甾烷素"[常用字段:智能] OR "谷甾醇"[常用字段:智能] OR "谷甾烷醇"[常用字段:智能] OR "豆甾醇"[常用字段:智能]) OR ("豆甾烷醇"[常用字段:智能] OR "油菜甾醇"[常用字段:智能])) AND ("高脂血症"[常用字段:智能] OR "高胆固醇血症"[常用字段:智能] OR "高甘油三酯血症"[常用字段:智能] OR "血脂异常"[常用字段:智能]) AND ("随机对照试验"[常用字段:智能] OR "随机"[常用字段:智能] OR "研究"[常用字段:智能])**

1. **For English databases, Pubmed was used as an example:**#1 AND #2 AND #3

#1 **(((((((((((phytosterols[MeSH Terms]) OR (phytosterol*[Title/Abstract])) OR (phytostanol*[Title/Abstract])) OR (plant sterol*[Title/Abstract])) OR (plant stanol*[Title/Abstract])) OR (sitosterol*[Title/Abstract])) OR (sitostanol*[Title/Abstract])) OR (campesterol*[Title/Abstract])) OR (campestanol*[Title/Abstract])) OR (stigmasterol*[Title/Abstract])) OR (stigmastanol*[Title/Abstract])) OR (brassicasterol*[Title/Abstract])**

#2**(((Hyperlipemia[MeSH Terms]) OR (Hypercholesterolemia[Title/Abstract])) OR (hyperlipoproteinemia[Title/Abstract])) OR (dyslipidemias[Title/Abstract])**

#3 **(((randomized controlled trial[Title/Abstract]) OR (RCT[Title/Abstract])) OR (random[Title/Abstract])) OR (stud*[Title/Abstract])**

1. **For English databases, Embase was used as an example:** #1 AND #2 AND #3

#1 ：

**'hyperlipemia'/exp OR hyperlipemia OR hypercholesterolemia:ab,ti OR hyperlipoproteinemia:ab,ti OR dyslipidemias:ab,ti**

#2：

**phytosterol* OR phytostanol*:ab,ti OR 'plant sterol*':ab,ti OR 'plant stanol*':ab,ti OR sitosterol*:ab,ti OR sitostanol*:ab,ti OR campesterol*:ab,ti OR campestanol*:ab,ti OR stigmasterol*:ab,ti OR stigmastanol*:ab,ti OR brassicasterol*:ab,ti**

#3：

**'randomized controlled trial'/exp OR 'randomized controlled trial' OR (randomized AND controlled AND ('trial'/exp OR trial)) OR rct:ab,ti**

1. **For English databases, Web of science was used as an example:** #1 AND #2

#1：

**TS=Hyperlipidemias OR TS=Hypercholesterolemia OR TS=hyperlipoproteinemia OR TS=dyslipidemias**

#2：

**TS=phytosterol* OR TS=phytostanol* OR TS=plant sterol OR TS=plant stanol* OR TS=sitosterol* OR TS=sitostanol* OR TS=campesterol* OR TS=campestanol* OR TS=stigmasterol* OR TS=stigmastanol* OR TS=brassicasterol***

#3：

**TS=randomized controlled trial OR TS=randomized OR TS=stud* OR TS=rct**

1. **For English databases, The Cochrane library was used as an example:** #1 AND #2 AND #3

#1：

**((phytosterol*):ti,ab,kw OR (phytostanol*):ti,ab,kw OR (plant sterol):ti,ab,kw OR (plant stanol*):ti,ab,kw OR (sitosterol*):ti,ab,kw OR (sitostanol*):ti,ab,kw OR (campesterol*):ti,ab,kw OR (campestanol*):ti,ab,kw OR (stigmasterol*):ti,ab,kw OR (stigmastanol*):ti,ab,kw OR (brassicasterol*):ti,ab,kw )**

#2：

**((Hyperlipemia):ti,ab,kw OR (Hypercholesterolemia):ti,ab,kw OR (hyperlipoproteinemia):ti,ab,kw OR (dyslipidemias):ti,ab,kw)**

#3：

**((randomized controlled trial):ti,ab,kw OR (randomized):ti,ab,kw OR (stud*):ti,ab,kw OR (rct):ti,ab,kw)**

1. **For English databases, The Scoups was used as an example:** #1 AND #2 AND #3

#1：

**( TITLE-ABS-KEY ( "phytosterol*" ) OR TITLE-ABS-KEY ( "phytostanol*" ) OR TITLE-ABS-KEY ( "plant sterol*" ) OR TITLE-ABS-KEY ( "plant stanol*" ) OR TITLE-ABS-KEY ( "sitosterol*" ) OR TITLE-ABS-KEY ( "sitostanol*" ) OR TITLE-ABS-KEY ( "campesterol*" ) OR TITLE-ABS-KEY ( "campestanol*" ) OR TITLE-ABS-KEY ( "stigmasterol*" ) OR TITLE-ABS-KEY ( "stigmastanol***

**" ) OR TITLE-ABS-KEY ( " brassicasterol*"))**

#2：

**( TITLE-ABS-KEY ( "Hyperlipemia" ) OR TITLE-ABS-KEY ( "Hypercholesterolemia" ) OR TITLE-ABS-KEY ( "hyperlipoproteinemia" ) OR TITLE-ABS-KEY ( "dyslipidemias" ))**

#3：

**( TITLE-ABS-KEY ( "randomized controlled trial" ) OR TITLE-ABS-KEY ( "randomized" ) OR TITLE-ABS-KEY ( "stud*" ) OR TITLE-ABS-KEY ( "RCT" ))**
